# Wogonin Attenuates Isoprenaline-Induced Myocardial Hypertrophy in Mice by Suppressing the PI3K/Akt Pathway

**DOI:** 10.3389/fphar.2018.00896

**Published:** 2018-08-13

**Authors:** Weichun Qian, Dongsheng Yu, Jia Zhang, Qiaoyun Hu, Chuanfeng Tang, Peiyu Liu, Peng Ye, Xiaoli Wang, Qiu Lv, Minglong Chen, Liang Sheng

**Affiliations:** ^1^Department of Cardiology, Nanjing First Hospital, Nanjing Medical University, Nanjing, China; ^2^Department of Pharmacology, School of Basic Medical Science, Nanjing Medical University, Nanjing, China; ^3^Department of Cardiology, The First Affiliated Hospital of Nanjing Medical University, Nanjing, China; ^4^Key Laboratory of Rare Metabolic Diseases, Nanjing Medical University, Nanjing, China; ^5^Neuroprotective Drug Discovery Key Laboratory, Nanjing Medical University, Nanjing, China

**Keywords:** wogonin, myocardial hypertrophy, Pik3ca, Nedd4l, ubiqutination

## Abstract

Many studies have focused on identifying therapeutic targets of myocardial hypertrophy for the treatment of correlative cardiac events. Wogonin is a natural flavonoid compound that displays a potent anti-hypertrophic effect. Knowledge of its pharmacological mechanisms might reveal an effective way to search for therapeutic targets. Myocardial hypertrophy was replicated by the subcutaneous implantation of an isoprenaline mini-pump in mice or isoprenaline treatment of H9C2 cells. Pathologic changes in cardiac structure were assessed by echocardiographic and histological examinations. The signaling transduction in hypertrophy-promoting pathways and the genes involved were detected by western blot and RT-qPCR. Wogonin significantly attenuated isoprenaline-induced myocardial hypertrophy *in vivo* and *in vitro* by suppressing phosphatidylinositol 3-kinase/protein kinase B (PI3K/Akt) hypertrophy-promoting pathway. Wogonin promoted the ubiquitination and degradation of PI3K catalytic subunit alpha (Pik3ca), the catalytic subunit of PI3K, which was upregulated by isoprenaline treatment. Wogonin also increased the expression of neural precursor cells expressing developmentally down-regulated gene 4-like (Nedd4l), the ubiquitin E3 ligase of Pik3ca. Therefore, wogonin targets Nedd4l to induce the degradation of Pik3ca, which reverses the over-activation of the PI3K/Akt pathway and consequently relieves the isoprenaline-induced myocardial hypertrophy.

## Introduction

Myocardial hypertrophy is characterized by thickening of the ventricle wall in the heart, an adaptive response to, for example, mechanical and neurohumoral stimulations (Hill and Olson, 2008; Maillet et al., 2013). At the early stage of myocardial hypertrophy, cardiomyocytes grow in length and/or in width, and it is the main reason for ventricular thickening (Francis et al., 1993; Shimizu and Minamino, 2016). However, if the stimuli persist, apoptosis of cardiomyocytes would occur and lead to heart failure, arrhythmia, and sudden death (Lyon et al., 2015). Thus myocardial hypertrophy is considered as the pathological foundation for multiple cardiac events (Haider et al., 1998).

β-adrenoceptors, locating on the membranes of the three major cardiac cell types (cardiomyocytes, fibroblasts, and endothelial cells), belong to the G protein-coupled receptor superfamily (Kawano et al., 2009). Their stimulation activates downstream signaling pathways that regulate different intracellular, sarcolemmal, and myofibrillar substrates (Feldman et al., 2005; Siryk-Bathgate et al., 2013). Neurohumoral stimulation or binding of catecholamine to β-adrenoceptors of cardiomyocytes causes the related heterotrimeric G proteins to dissociate into G_αs_/G_βγ_ and G_αi_/G_βγ_ subunits (Nienaber et al., 2003). G_αs_ activates adenylyl cyclase to generate the second messenger cAMP, leading to increased heart rate and myocardiac contractility (Kamide et al., 2015). The G_αi_ subunit activates the PI3K/Akt and mitogen-activated protein kinase (MAPK) signaling pathways both promoting myocardial hypertrophy (Esposito et al., 2002; Lohse et al., 2003; Feldman et al., 2005; Heineke and Molkentin, 2006; Go et al., 2014). Therefore, β-adrenoceptor signaling pathway should likely contain some potential targets for myocardial hypertrophy therapy.

**FIGURE 1:**
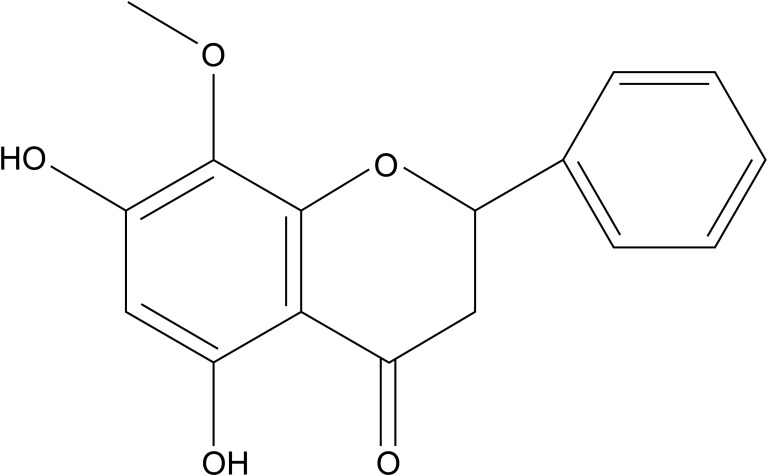
Chemical structure of wogonin (5,7-dihydroxy-8-methoxyflavone).

Wogonin (5,7-dihydroxy-8-methoxyflavone; **Figure 1**) is a natural dihydroxyl flavonoid compound isolated from the roots of *Scutellaria baicalensi Georg, S. amoena C. H. Wright*, or *S. rivularis Wall* (Tai et al., 2005). It has a variety of biological activities, including anti-oxidation, anti-inflammation, neuroprotection, and anti-carcinoma activities (Liu et al., 2011; Chirumbolo, 2013; Ku and Bae, 2015). Wogonin reportedly attenuates diabetic cardiomyopathy (Khan et al., 2016). However, whether and how wogonin attenuates β-adrenoceptor-mediated myocardial hypertrophy is unknown.

In the present study, we confirm the therapeutic effect of wogonin on isoprenaline-induced myocardial hypertrophy and identify Nedd41 as the target of wogonin. Nedd4l is a ubiquitin E3 ligase that promotes the degradation of Pik3ca and thus attenuates the over-activation of the PI3K/Akt pathway stimulated by isoprenaline treatment.

## Materials and Methods

### Materials and Reagents

Wogonin was purchased from Spring & Autumn Biotec Co., Ltd. (Nanjing, China). Isoprenaline was purchased from Tokyo Chemical Industry Co., Ltd. (Tokyo, Japan). All-trans-retinoic acid (RA), DBcAMP, and phorbol 12, 13-dibutyrate (PDBU) were purchased from Sigma-Aldrich LLC. (Shanghai, China). MG132 was obtained from Selleck Chemical (Houston, TX, United States). The vectors pUSEamp(+)/myc-tagged Akt (constitutively active, CA) and pUSEamp(+)-Pik3ca were kindly provided by Liangyou Rui from the University of Michigan. The vectors pcDNA3HA and pGL3-Basic were provided by Dongping Wei from Nanjing First Hospital. The empty vector pAdeno-MCMV-MCS-3Flag and pDONR223 vector carrying a human Nedd4l gene were purchased from Obio Technology Corp., Ltd. (Shanghai, China) and Public Protein/Plasmid Library (Nanjing, China), respectively. The primers 5′-TCGAGCTCAAGCTTCGAATTCATGGAGCGACCCTATACATTT-3′ and 5′-GTCATCCTTGTAGTCGGATCCATCCACCCCTTCAAATCCTT-3′ were used to subclone human Nedd4l cDNA from pDONR223-Nedd4l by PCR. The PCR products were recombined with pAdeno-MCMV-MCS-3Flag vector cut by EcoR1/BamH1 to obtain the expression vector pAdeno-MCMV-MCS-3Flag-Nedd4l. Pik3ca cDNA was subcloned from pUSEamp(+)-Pik3ca using the primers 5′-GATCCCCCGGGCTGCAGGAATTCATGGGGAGCAGCAAGAGCAAG-3′ and 5′-ATAGAATAGGGCCCCCCCTCGAGTCAGTTCAAAGCATGCTG-3′. The PCR products were recombined with pcDNA3HA vector cut by EcoR1/Xho1 to obtain the expression vector pcDNA3HA-Pik3ca.

### Animals and Treatment

**FIGURE 2:**
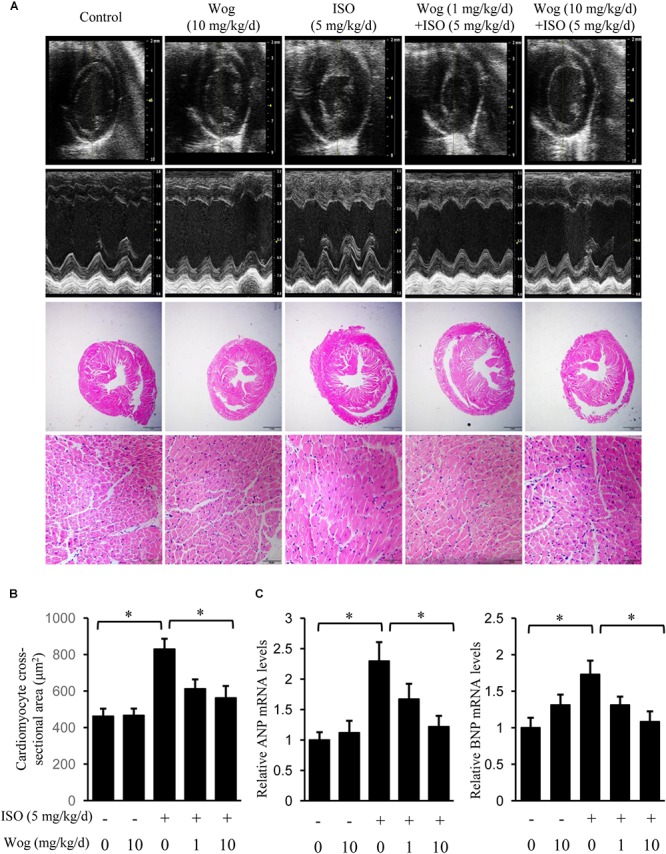
Wogonin attenuates isoprenaline-induced myocardial hypertrophy in mice. Male 8-week-old ICR mice were subcutaneously implanted with mini-pumps delivering isoprenaline (ISO, 5 mg/kg/day, *n* = 6) or PBS (Control, *n* = 5), and then treated by wogonin (Wog, 1 mg, 10 mg kg/day, *n* = 6) via intraperitoneal injection for 14 days. **(A)** Representative echocardiography and H&E staining in a cross-section of the heart. **(B)** Quantitation of cross-sectional area of cardiomyocytes (*n* = 8). **(C)** The mRNA levels of ANP and BNP in heart were determined using RT-qPCR and normalized to 36/B4. Data are expressed as fold-change relative to the level of control group are presented as the mean ± SEM; ^∗^*p* < 0.05, versus control or ISO alone treated group.

Male ICR mice were purchased from Model Animal Research Center of Nanjing University. They were housed in a pathogen-free barrier facility with a 12-h light/dark cycle and given free access to food and water. Eight-week-old mice (*n* = 29) were divided into five groups as indicated (**Figure 2**). Under inhalation anesthesia by isoflurane, an osmotic minipump (model 2004D; Alzet, Durect Corp, Cupertino, CA, United States) was implanted subcutaneously on the back of the neck (Krenek et al., 2009; Heap et al., 2015) and the delivery of isoprenaline (5 mg /kg/day) was started. Immediately after implantation, wogonin was administered by intraperitoneal injection for 2 weeks at a dose of 1 mg/kg or 10 mg/kg once daily. All animal experiments complied with the guidelines of the Nanjing Medical University Regulations of Animal Experiments and were approved by the Animal Experiment Committee of the Nanjing Medical University.

### Cell Culture and Treatment

Rat fetal cardiomyocytes (H9c2) were obtained from American Type Collection (ATCC, Manassas, VA, United States). H9c2 cells were cultured in Dulbecco’s modified Eagle’s high-glucose medium (DMEM) supplemented with 10% fetal bovine serum (FBS) and 1% penicillin/streptomycin (Hyclone) in a humidified incubator in an atmosphere of 5% CO_2_ at 37°C. When the cell confluence reached to 70–80%, cell culture medium was switched to DMEM containing 1% FBS and 1 μM RA for further 5-day culture before experimental treatments.

### Echocardiographic Assessments

After the 2-week medication, mice were anesthetized with isoflurane for echocardiographic examination. The images were obtained using a Vevo 2100 system with a 45 MHz probe (Visualsonics, Toronto, ON, Canada) to evaluate the cardiac function and chamber size. M-mode tracings were used to measure interventricular septum diameter (IVSd), left ventricular posterior wall diameter (LVPWd), left ventricular end-diastolic diameter (LVIDd), and left ventricular end-systolic diameter (LVIDs). Ejection fraction (EF, %) and left ventricular (LV) mass were calculated as left ventricular end-systolic volume (LVESV) = 7.0/(2.4 + LVIDs)^∗^LVIDs^3^; left ventricular end-diastolic volume (LVEDV) = 7.0/(2.4 + LVIDd)^∗^ LVIDd^3^. EF = 100^∗^((LVEDV-LVESV)/LVEDV). LV Mass = 1.053^∗^((LVIDd+LVPWd+IVSd)^3^-LVIDd^3^).

### Histological Analysis

Mouse hearts were fixed in 4% paraformaldehyde solution for 48 h, dehydrated in ascending grades of ethanol, and embedded in wax. Heart sections were prepared for routine haematoxylin and eosin (H&E) staining. H9c2 cells were grown on the glass coverslips for immunofluorescence staining. Cells were washed twice with PBS, fixed for 10 min in 4% paraformaldehyde solution, and permeabilized with 0.1% Triton X-100 plus 1% bovine serum albumin (BSA) for 1 h. Then the cells were incubated with alpha-smooth muscle actin (α-SMA) antibody (1:100 dilution) for 4 h at 37°C followed by staining with a 1:100 dilution of 4′,6-diamidino-2-phenylindole (DAPI) for 10 min. A model IX72 fluorescence inverted microscope (Olympus, Tokyo, Japan) was used to observe the cells. α-SMA antibody was purchased from Abcam (Cambridge, United Kingdom). Antibody against DAPI was purchased from Sigma-Aldrich (St. Louis, MO, United States).

### Real Time Quantitative Polymerase Chain Reaction (RT-qPCR)

Total RNA extracted from mouse heart tissues and H9c2 cells were reverse-transcribed as previously described (Chen et al., 2016). qPCR was performed using SYBR Premix Master Mix (Thermo Fisher Scientific Inc., Shanghai, China). Relative mRNA levels of target genes were quantified using comparative threshold (CT) normalized to house-keeping genes [ribosomal protein, large, P0 (36B4) for mouse heart or β-actin for H9c2 cells]. The primer sequences used for qPCR are demonstrated in **Table 1**.

**Table 1:** Primer sequences used for quantitative RT-qPCR.

Target gene	Forward primers (5′–3′)	Reverse primers (5′–3′)
ANP (mouse)	GCTTCCAGGCCATATTGGAG	GGGGGCATGACCTCATCTT
BNP (mouse)	GAGGTCACTCCTATCCTCTGG	GCCATTTCCTCCGACTTTTCTC
ANP (rat)	GCAAACATCAGATCGTGCCC	TTGCTCCAATATGGCCTGGG
BNP (rat)	TAGGTCTCAAGACAGCGCCT	CGCCGATCCGGTCTATCTTC
Pik3ca(rat)	CCACGACCATCTTCGGGTG	ACGGAGGCATTCTAAAGTCACTA
Nedd4l(rat)	GGCACTTTACGGAGGTCACA	GAGGCCAAGTTCACGACTGA
Tnnc1(rat)	CTGTCGGATCTCTTCCGCAT	TTCCGTGATGGTCTCACCTG
Tnnt2(rat)	CCTGACGGAGAGAGAGTGGA	TGTTCTCGAAGTGAGCCTCG
36/B4(mouse)	AAGCGCGTCCTGGCATTGTCT	CCGCAGGGGCAGCAGTGG
β-actin(rat)	CCCGCGAGTACAACCTTCTT	CGCAGCGATATCGTCATCCA


### Immunoblotting

As previously described (Chen et al., 2016), extracts from mouse hearts or cells were immunoblotted using primary antibodies against Nedd4l, Pik3ca, p-Akt, Akt, cAMP response elements binding (CREB), phospho (p)-CREB, c-jun N-terminal kinase (JNK), p-JNK, p-p38, p38, extracellular signal-regulated kinase 1/2 (ERK1/2), p-ERK1/2, forkhead box protein O1 (FoxO1), p-FoxO1, FoxO3a, p-FoxO3a, ubiquitin, G-protein-coupled receptor kinase 2 (GRK2), and β-actin (all from Cell Signaling Technologies, Beverly, MA, United States). Blots were incubated with secondary antibody conjugated with horseradish peroxidase (Cell Signaling Technologies) and visualized using an ECL kit (Millipore, Billerica, MA, United States). The chemiluminiscence signals were detected by a model 4200SF device (Tanon Shanghai, China).

### Luciferase Assay

The -2000 to +1 promoter region of human Nedd4l was amplified by PCR with genomic DNA from HepG2 cells using primers 5′-GGTACCGAGCTCTTACGCGTAGGTAGAGAAGCACTGACTCC-3′ and 5′-CTTAGATCGCAGATCTCGAGCGGCCGGGCTTTCC-3′. The PCR products were recombined with pGL3-Basic vector cut by Mlu1/Xho1 to obtain the reporter construct pGL3–2000/+1LUC. H9c2 cells were transfected with luciferase reporter plasmids plus the internal control vector pRL-TK-Renilla for luciferase assays in 24-well plates. After an overnight culture, cells were incubated for 24 h in complete medium supplemented with wogonin (10 μM). Luciferase activity was measured using the dual-luciferase reporter assay system (Promega, Madison, WI, United States) and normalized to Renilla luciferase.

### Statistical Analyses

The data and statistical analyses in this study comply with the recommendations on experimental design and analysis in pharmacology (Curtis et al., 2015). Data are presented as means ± SE. Data between groups were analyzed by Student’s *t*-test or one-way ANOVA followed by LSD–Dunn multiple comparisons. *p* < 0.05 was considered statistically significant.

## Results

### Wogonin Attenuates Isoprenaline-Induced Myocardial Hypertrophy in Mice

To evaluate the anti-hypertrophic effect of wogonin, myocardial hypertrophy in mice was replicated by subcutaneous implantation of mini-pump delivering isoprenaline for 14 days. Wogonin failed to prevent the isoprenaline-mediated reduction in body weight (BW) but did reverse the abnormal increase in heart weight (HW) (**Table 2** and **Figure 2A**). Wogonin alone had no effects on BW or HW. The detailed anatomical changes in the heart were detected by echocardiography and reflected via hypertrophic indexes including HW, HW/BW, IVSd, LVPWd, LV Mass, LVIDd, and LVIDs. EF is to evaluate the left ventricular systolic function. Isoprenaline treatment increased HW, HW/BW, IVSd, LVPWd, and LV Mass but decreased LVIDd and LVIDs. Those changes, showing typical for concentric hypertrophy, were all reversed by wogonin. EF was neither worsened by isoprenaline nor affected by wogonin. Histologic analysis of heart section slices also revealed that wogonin attenuated the enlargement in heart size after isoprenaline treatment (**Figures 2A,B**). Atrial natriuretic peptide (ANP) and brain natriuretic peptide (BNP) are two well-known natriuretic peptides secreted by cardiac muscle cells when hypertrophy occurs (Vasan, 2006). Thus, the expressions of ANP and BNP in heart were determined as the important biomarkers for myocardial hypertrophy. As shown in **Figure 2C**, wogonin relieved isoprenaline-induced increase in mRNA levels of ANP and BNP in a dose-dependent manner but wogonin alone did not affect their expression.

**Table 2:** Effect of wogonin on heart weight and thickness.

Parameter	Control (*n* = 5)	Wog (10 mg/kg) (*n* = 6)	ISO (*n* = 6)	ISO + Wog (1 mg/kg) (*n* = 6)	ISO + Wog (10 mg/kg) (*n* = 6)
BW(g)	35.20 ± 0.67	35.25 ± 0.25	31.93 ± 0.51*	32.03 ± 0.31*	31.27 ± 0.82*
HW(mg)	128.00 ± 2.65	125.6 ± 1.08	138.17 ± 2.27*	133.33 ± 0.80	121.50 ± 2.23#
HW/BW	3.64 ± 0.09	3.57 ± 0.06	4.33 ± 0.04*	4.16 ± 0.05	3.89 ± 0.09#
IVSd (mm)	0.72 ± 0.02	0.69 ± 0.01	0.89 ± 0.02*	0.83 ± 0.02#	0.76 ± 0.02#
LVPWd (mm)	0.75 ± 0.01	0.76 ± 0.01	0.88 ± 0.03*	0.83 ± 0.04	0.81 ± 0.02#
LVMass (mg)	100.22 ± 2.07	99.43 ± 1.35	111.07 ± 2.13*	105.67 ± 3.58	103.21 ± 3.11#
LVIDd (mm)	3.87 ± 0.05	3.90 ± 0.43	3.54 ± 0.07*	3.63 ± 0.03	3.74 ± 0.07#
LVIDs (mm)	2.67 ± 0.08	2.56 ± 0.02	2.35 ± 0.04*	2.40 ± 0.03	2.53 ± 0.08
EF(%)	59.61 ± 2.30	58.35 ± 0.91	62.84 ± 1.99	60.47 ± 1.10	61.22 ± 1.43


*All data are presented as mean ± SEM. BW, body weight; HW, heart weight; HW/BW, body weight/heart weight; IVSd, interventricular septum diameter; LVPWd, left ventricular posterior wall diameter; LV Mass, left ventricular mass; LVIDd, left ventricular end-diastolic diameter; LVIDs, left ventricular end-systolic diameter. EF (%), Ejection fraction. versus Control ^∗^*P* < 0.05; versus ISO ^#^*P* < 0.05.*

### Wogonin Reverses Isoprenaline-Induced Hypertrophy in RA Differentiated H9c2 Cells

**FIGURE 3:**
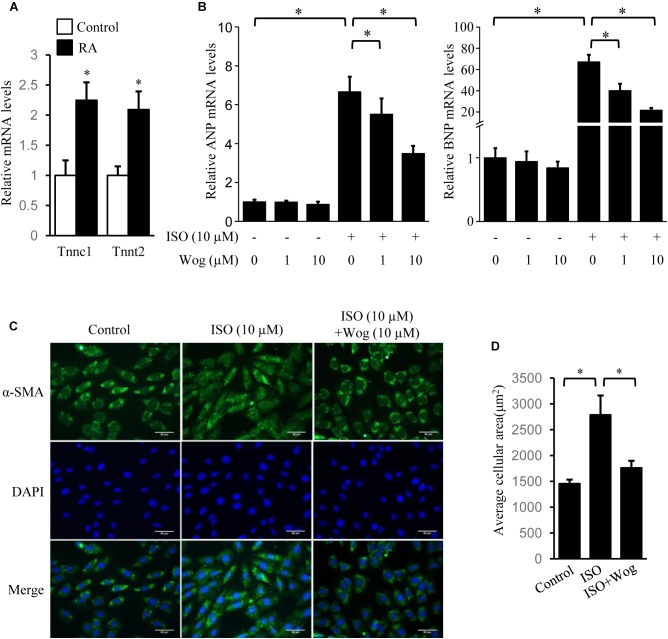
Wogonin reverses isoprenaline-induced hypertrophy in RA-differentiated H9c2 cells. H9c2 cells were cultured in DMEM containing 1% FBS and 1 μM RA for 5 days before incubating with wogonin (1 and 10 μM) with/without isoprenaline (10 μM) for a further 24 h. **(A)** The cardiac-specific genes, Tnnc1 and Tnnt2 (*n* = 3), and **(B)** myocardial cell-specific hypertrophic markers, ANP and BNP (*n* = 5), were determined with RT-qPCR and normalized to β-actin. Data are expressed as fold-change relative to the level of control cells. **(C)** H9c2 cell shape was demonstrated by the immunofluorescence with α-SMA antibody. **(D)** Quantitation of average cell surface area (*n* = 8). Data are presented as mean ± SEM; ^∗^*p* < 0.05 versus control.

The H9c2 cell line was originally derived from embryonic rat ventricular tissue. These cells are widely used as an *in vitro* tool to investigate pathomechanism of cardiomyocytes, including cardiac hypertrophy (Watkins et al., 2011). H9c2 cells are undifferentiated myoblasts. The addition of RA drives cell differentiation toward a cardiac phenotype (Branco et al., 2015). The increased mRNA levels of Tnnc1 and Tnnt2, the cardiac-specific genes, indicated the success in cell differentiation (**Figure 3A**). Myocardial hypertrophy was replicated by isoprenaline treatment in differentiated H9c2 cells. The mRNA levels of ANP and BNP were also investigated as hypertrophic markers. Compared to the dose of 1 μM, wogonin (10 μM) more effectively reduced the cellular mRNA levels of ANP and BNP which had been enhanced by isoprenaline treatment (**Figure 3B**). In cardiomyocytes, α-SMA is a major constituent of the cytoskeleton, which determines the cell shape. Thus, staining of α-SMA was performed to observe the morphology of H9c2 cells. The average area of H9c2 cells was significantly expanded by isoprenaline treatment, while wogonin relieved this effect (**Figures 3C,D**).

### Wogonin Represses Akt Signaling Pathway Initiated by Isoprenaline Treatment

**FIGURE 4:**
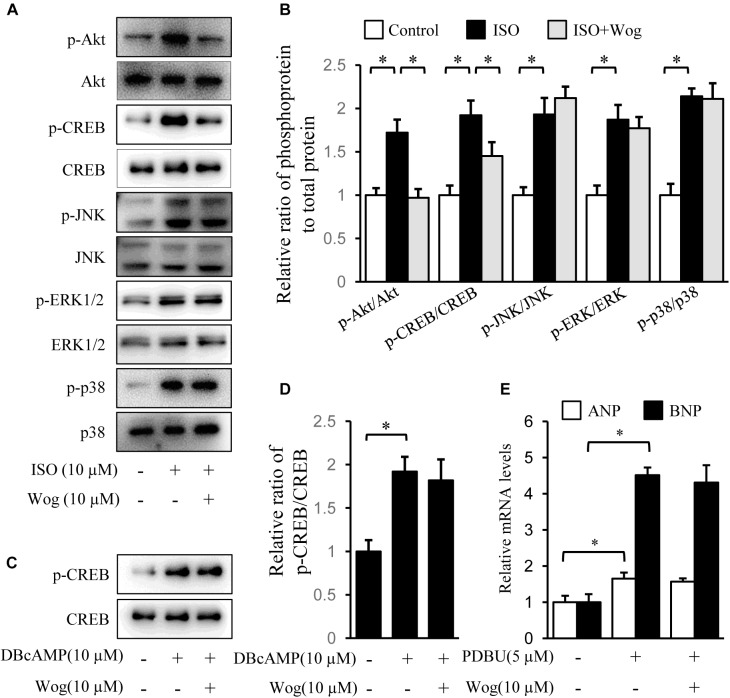
Wogonin inhibits the Akt signaling pathway initiated by isoprenaline treatment. H9c2 cells were cultured in DMEM containing 1% FBS and 1 μM RA for 5-day differentiation. **(A)** Differentiated H9c2 cells were treated with isoprenaline (10 μM) and/or wogonin (10 μM) with indicated dose for 24 h. Cell extracts were blotted by antibodies against pAkt, Akt, pCREB, CREB, pJNK, JNK, pERK1/2, ERK1/2, pP38 and P38. **(B)** Quantitation of phosphoprotein to total protein (*n* = 3). **(C)** Differentiated H9c2 cells, pretreated with wogonin (10 μM) for 15 min, then were incubated with DBcAMP (10 μM) for another 15 min. Cell extracts were blotted by antibodies against p-CREB and CREB. **(D)** Quantitation of p-CREB to CREB (*n* = 3). **(E)** Differentiated H9c2 cells were treated with wogonin (10 μM) and PDBU (5 μM) for 24 h. The mRNA levels of ANP and BNP were determined by RT-qPCR (*n* = 5). Data are given as mean ± SEM; ^∗^*p* < 0.05 versus control.

Isoprenaline induces cardiomyocyte hypertrophy via multiple pathways, including PI3K/Akt, cAMP/PKA/CREB, MAPKs (JNK, p38, and ERK), and PKC (Heineke and Molkentin, 2006). The phosphorylation levels of Akt, CREB, JNK, p38, and ERK were significantly increased by isoprenaline. Wogonin reversed the phosphorylation levels of Akt and CREB (**Figures 4A,B**). CREB phosphorylation was mediated by two pathways, including cAMP/PKA and PI3K/Akt, during isoprenaline treatment (Bullock and Habener, 1998; Khalilimeybodi et al., 2017). Wogonin did not affect CREB phosphorylation induced by dibutyl cyclic adenosine acid (DB-cAMP), the analog of cAMP (**Figures 4C,D**), implying that wogonin reduces CREB phosphorylation via the PI3K/Akt pathway instead of the cAMP/PKA pathway. PKCs were activated by its selective agonist, PDBU, which increased the mRNA levels of ANP and BNP. However, wogonin did not affect PKC-induced transcription of ANP and BNP, indicating that the anti-hypertrophy effect of wogonin is not involved in the PKC pathway (**Figure 4E**).

### Wogonin Improves Myocardial Hypertrophy by Reducing Pik3ca Expression

**FIGURE 5:**
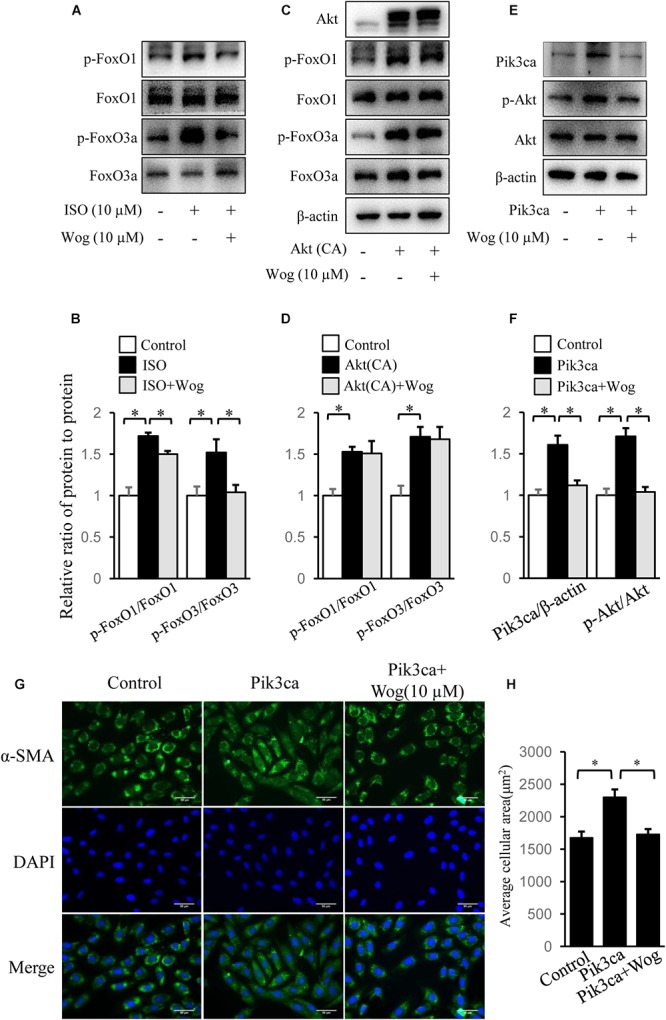
Wogonin improves myocardial hypertrophy by downregulating Pik3ca**. (A)** Differentiated H9c2 cells were treated with isoprenaline (10 μM) and/or wogonin (10 μM) for 24 h. Cell extracts were blotted by antibodies against p-FoxO1, FoxO1, p-FoxO3a and FoxO3a. H9c2 cells were transfected with pUSEamp(+)-Akt(CA) **(C)** or pUSEamp(+)-Pik3ca **(E)** and grew for 48 h. Then wogonin (10 μM) was added 24 h before harvesting. Cell extracts were immunobloted by indicated antibodies. **(B,D,F)** Quantitation of indicated protein bands (*n* = 3). **(G)** H9c2 cell shape was demonstrated by the immunofluorescence with α-SMA antibody. **(H)** Quantitation of average cell surface area (*n* = 8). Data are presented as mean ± SEM; ^∗^*p* < 0.05.

FoxO1, FoxO3a, and CREB mediate PI3K/Akt pathway-induced gene expression involved in myocardial hypertrophy (Ronnebaum and Patterson, 2010; Khalilimeybodi et al., 2017). We observed that isoprenaline increased the phosphorylation levels of Akt, and the downstream FoxO1, FoxO3a, and CREB, which were all inhibited by wogonin (**Figures 4A,B, 5A,B**). Thus, we reasoned that wogonin specifically acts on the PI3K/Akt signaling pathway. The fact that constitutively active Akt [Akt (CA)] transfected into H9c2 cells significantly enhanced the phosphorylation of FoxO1 and FoxO3a, which were not affected by wogonin, implies that the target of wogonin is located in the upstream of Akt (**Figures 5C,D**). As class IA PI3K, one of three types of PI3Ks, plays an important role in cardiac growth and function (Shioi et al., 2000), we overexpressed Pik3ca, the catalytic subunit of class IA PI3K, into H9c2 cells to initiate the phosphorylation of Akt. Interestingly, wogonin reversed the Akt phosphorylation by reducing the level of Pik3ca protein (**Figures 5E,F**). Consistently, α-SMA staining showed that Pik3ca overexpression amplified the size of H9c2 cells and wogonin reversed this pathological change (**Figures 5G,H**). When catecholamine binding to β-adrenoceptors, the G protein–coupled receptor kinase-2 (GRK2) mediates the translocation of PI3K to β-adrenoceptors and then enhances the recruitment of β-arrestin and AP-2, which finally results in the internalization and downregulation of β-adrenoceptors (Naga Prasad et al., 2002). It has reported that disrupts the interaction between PI3K and GRK2 by displacing class I PI3K isoforms blocks agonist-stimulated β-adrenoceptors internalization (Nienaber et al., 2003). To test whether wogonin potentially affects β-adrenoceptor function by modulating the interaction between PI3K and GRK2, precipitation was performed. As shown in **Supplementary Figure S1**, wogonin treatment reduced the binding of GRK2 to Pik3ca in H9c2 cells, mostly due to wogonin-induced reduction in Pik3ca expression.

### Wogonin Induces Pik3ca Degradation by Promoting Its Ubiquitination

**FIGURE 6:**
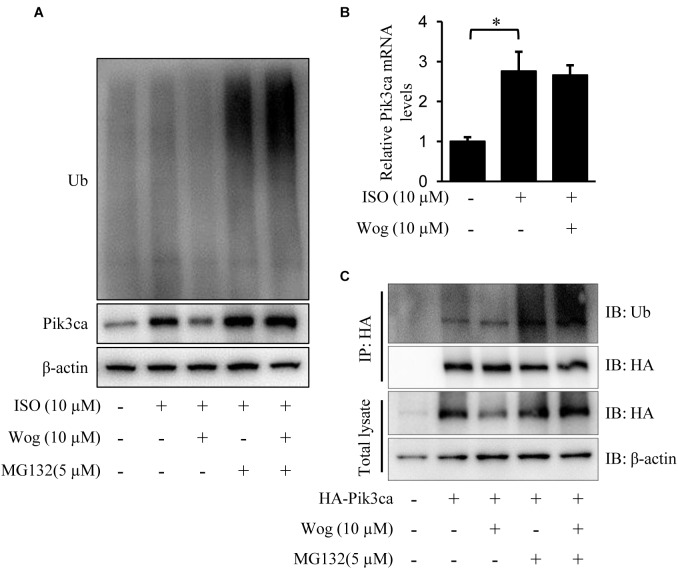
Wogonin induces degradation of Pik3ca by promoting its ubiqutination. **(A,B)** Differentiated H9c2 cells were treated with isoprenaline (10 μM) and/or wogonin (10 μM) for 24 h. **(A)** Cells were treated by MG132 (5 μM) for 8 h before harvesting. Cell extracts were immunoblotted by ubiquitin, Pik3ca, and β-actin antibodies. **(B)** The mRNA level of Pik3ca was determined by RT-qPCR and normalized to β-actin. **(C)** H9c2 cells over-expressing HA-tagged Pik3ca by transfection of pcDNA3HA-Pik3ca were treated with wogonin (10 μM, 24 h) and MG132 (5 μM, 8 h) in sequence. Cell extracts were immunoprecipitated by HA-beads and immunoblotted with antibodies against ubiquitin and HA. Data are presented as the mean ± SEM; ^∗^*p* < 0.05, versus control.

Pik3ca was significantly increased at the protein and mRNA levels in H9c2 cells with isoprenaline treatment (**Figures 6A,B**). Wogonin reduced the protein level of Pik3ca but failed to decrease its mRNA level (**Figures 6A,B**). We reasoned that wogonin accelerates the degradation of Pik3ca protein. The classic protein degradation process depends on the ubiquitination-proteasome system, which includes protein-ubiquitination by ubiquitin ligase and subsequent protein-recognization by proteasomes for retrogradation. Thus, we utilized the proteasome inhibitor MG132 to prevent the ubiquitinated proteins from proteasome-mediated degradation. Wogonin induced an enhancement in the ubiquitination of plenty of proteins, which should include Pik3ca, since MG132 reversed the wogonin-induced downregulation of Pik3ca (**Figure 6A**). To confirm the specificity of the wogonin-mediated ubiquitination of Pik3ca, hemaglutinin (HA)-tagged Pik3ca was transfected into H9c2 cells. The cells were then treated with wogonin and MG132. The original protein amounts of HA-tagged Pik3ca were equal in each condition and immunoprecipitation was easier to operate with HA-tagged beads. As shown in **Figure 6C**, the precipitated Pik3ca displayed higher ubiquitination levels after wogonin treatment.

### Wogonin Enhancement of Nedd41 Expression

**FIGURE 7:**
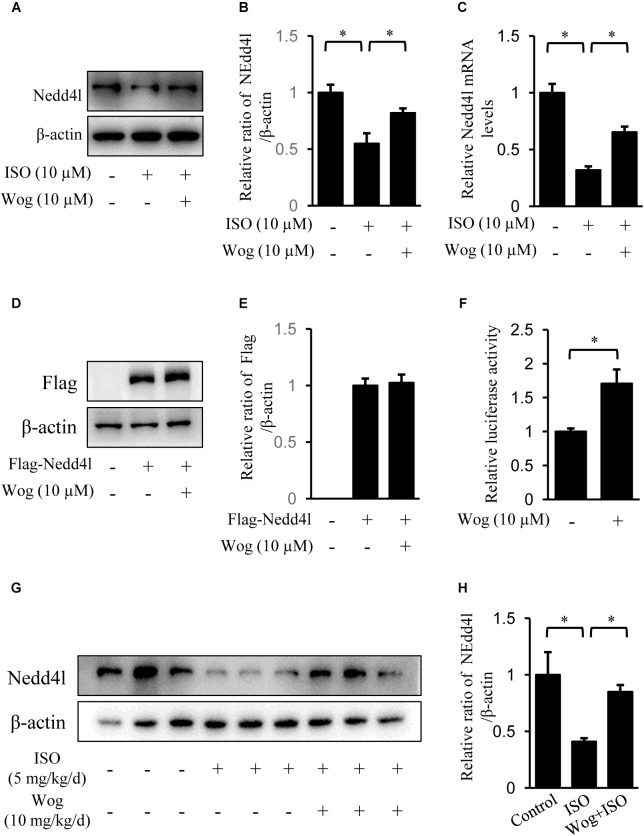
Wogonin enhances the transcription of Nedd4l. **(A–C)** Differentiated H9c2 cells were treated with isoprenaline (10 μM) and/or wogonin (10 μM) for 24 h. The protein and mRNA levels of Nedd4l were determined by immunoblot and RT-qPCR. **(D)** H9c2 cells were transfected with pAdeno-MCMV-MCS-3Flag-Nedd4l expression vector and grew for 48 h. Cells were treated with wogonin (10 μM) for another 24 h before harvesting. Cell extracts were immunoblotted by antibodies against Flag and β-actin. **(F)** H9c2 cells were transfected with luciferase construct containing Nedd4l promoter (–2000/+1) with wogonin (10 μM) treatment for 24 h. Nedd4l promoter driven luciferase activity was normalized to Renilla luciferase activity and expressed as fold-change relative to the level of control. **(G)** Protein level of Nedd4l in heart were determined with immunoblot. **(B,E,H)** Quantitation of indicated protein bands (*n* = 3). Data are presented as mean ± SEM; ^∗^*p* < 0.05, versus control.

Nedd4l is the specific ubiquitin ligase of Pik3ca, belonging to the Nedd4 (neural precursor cell-expressed developmentally down-regulated gene 4) family (Yang and Kumar, 2010; Wang Z. et al., 2016). Isoprenaline treatment significantly reduced the mRNA and protein levels of Nedd4l in H9c2 cells (**Figures 7A–C**), which led to the upregulation in protein level of Pik3ca (**Figure 6A**). Besides, isoprenaline also increased the mRNA level of Pik3ca (**Figure 6B**). Wogonin reversed the inhibitory effect of isoprenaline on Nedd4l expression at the transcriptional and post-transcriptional levels (**Figures 7A–C**). We expressed Flag-tagged Nedd4l in H9c2 cells by transient transfection and found that wogonin treatment did not affect the protein levels of exogenous Nedd4l (**Figures 7D,E**indicating that wogonin regulates Nedd4l expression only at the transcriptional level. This was confirmed in the luciferase assay, as wogonin significantly enhanced the Nedd4l promoter-driven luciferase activity (**Figure 7F**). In line with the data from cells, wogonin treatment significantly reversed the downregulation by isoprenaline on protein level of Nedd4l in mouse heart (**Figures 7G,H**).

## Discussion

The present study confirms the attenuation by wogonin in isoprenaline-induced myocardial hypertrophy *in vivo* and *in vitro*. It reminds us that wogonin not only shows its therapeutic value but also can function as a probe to search for new targets for myocardial hypertrophy therapy.

Sympathetic activation is a main native factor in the progression of myocardial hypertrophy, which generates catecholamine to activate β-adrenoceptors as well as its downstream signaling and thus induce hypertrophic gene expression (Ozakca et al., 2013; Tank and Lee, 2015). There are three subtypes of β-adrenoceptors: β1, β2 and β3. β1 and β2-adrenoceptors are the two major adrenoceptor types in the heart (Kawano et al., 2009). Once activated, β1 and β2-adrenoceptors both bind to G_αs_ subunits to activate the classical adenylate cyclase/cAMP/PKA pathway (Kamide et al., 2015) or bind G_αi_ to activate the non-classical PI3K/Akt pathway (Kitaura, 2013). As a β-adrenoceptor agonist, isoprenaline was used to replicate myocardial hypertrophic model in present study (Heap et al., 2015; Wang J.J. et al., 2016). Fourteen-day treatment leads to the typical concentric hypertrophy with still compensated left ventricular systolic function. Actually, we chose a hypertrophic model at the early phase of cardiac remodeling in order to explore the therapeutic effect of wogonin on early myocardial hypertrophy, as early intervention is much more effective in the therapy of myocardial hypertrophy, which would prevent the heart from apoptosis/fibrosis and further developing into heart failure. Once left ventricular systolic function is uncompensatory, it is too late to return to normal (Bloom et al., 2017). Therefore, early treatment of wogonin has more clinical significance and is hence more worth studying. Notably, early treatment of wogonin attenuated the development of concentric hypertrophy and decreased mRNA expression of the hypertrophic markers, ANP and BNP (**Figure 2**). We also replicated the hypertrophic model with isoprenaline in differentiated H9c2 cells and found that wogonin reduced the amplified size and hypertrophic mark gene expression in H9c2 cells (**Figure 3**). Thus, the antihypertrophic effect of wogonin may depend on the amelioration in the abnormal enlargement of cardiomyocytes.

As far as we know, there are several pathways mediating myocardial hypertrophy activated by β-adrenoceptors. They include the PI3K/Akt, adenylate cyclase/cAMP/PKA, MAPK, and PKC signaling pathways (Heineke and Molkentin, 2006; Khalilimeybodi et al., 2017). We tested the effect of wogonin on the signaling transduction and found that wogonin reduced the phosphorylation of Akt and CREB but had no effects on the MAPK or PKC pathways (**Figure 4**). Phosphorylation of CREB can be induced by Akt or PKA (Kato et al., 2007; Zhang et al., 2014). The fact that wogonin cannot inhibit DBcAMP-induced phosphorylation of CREB suggests that the reduction in CREB phosphorylation is the result of inhibition on Akt, rather than suppression of the cAMP/PKA pathway by wogonin (**Figure 4**). Therefore, wogonin exerts its antihypertrophic effect by suppressing PI3K/Akt pathway. The expression of ANP and BNP are also regulated by PI3K/Akt pathway through their transcriptional factor, NF-AT3, since activated Akt suppresses the downstream glycogen synthase kinase 3 beta (GSK3β), the kinase of NF-AT3, and thus provokes NF-AT3 by reducing its phosphorylation level (Redondo-Diéguez, 2000). Therefore, wogonin may at least reduce ANP and BNP transcription via PI3K/Akt pathway. However, whether other pathways regulating the expression of ANP and BNP, such as Ca^2+^/calcineurin/NF-AT3 (Molkentin et al., 1998; Redondo-Diéguez, 2000) and STAT3/glycoprotein 130 (Zhang et al., 2008), are affected by wogonin remains to be explored.

The PI3K/Akt pathway has been linked to an extraordinarily diverse group of cellular functions, including cell growth, proliferation, differentiation, motility, survival, and intracellular trafficking (Abeyrathna and Su, 2015). Activated Akt induces compensatory myocardial hypertrophy in physiological conditions (Latronico et al., 2010) or leads to uncompensatory myocardial hypertrophy in pathological conditions if the activation of Akt persists (Condorelli et al., 2002; Shiojima, 2005). Since wogonin reduced the phosphorylation level of Akt but could not terminate hypertrophic signaling transduction promoted by constitutively active Akt (**Figure 5**), we reason that the target of wogonin is located in the upstream of Akt.

Presumably, PI3K is a potential target of wogonin. Mammalian PI3K can be divided into three major classes (class I, II, and III) based on their structure and substrate specificity. The class I PI3K can be further divided into two subtypes, class IA and class IB. The mammalian class IA PI3Ks are heterodimers of a 110 kDa catalytic subunit (p110α, p110β, or p110δ) and a regulatory subunit of 85 or 55 kDa (p85/p55), whereas the class IB is composed of a p110γ catalytic subunit and a p101 regulatory subunit (Toker and Cantley, 1997; Aoyagi and Matsui, 2011). Class IA PI3Ks plays an important role in cardiac growth and hypertrophy (Crackower et al., 2002). Since the heterodimer, p110α/p85, is the dominant form of class IA PI3K (Yan et al., 2015), we overexpressed p110α (Pik3ca) to enhance the PI3K activity in H9c2 cells, and observed the enhancement in hypertrophic development, including amplification of cell size and activation of Akt (**Figure 5**). Importantly, wogonin reduced the protein level of Pik3ca and reversed the subsequent effects induced by Pik3ca overexpression, which confirms that wogonin targets PI3K. Interestingly, isoprenaline not only promotes myocardial hypertrophy via β-adrenoceptor-mediated activation of PI3K/Akt pathway, but also induces the hyperfunction of PI3K/Akt pathway through upregulating Pik3ca expression (**Figure 6**). Besides the contribution to myocardial hypertrophy, PI3K also induces internalization and downregulation of β-adrenoceptors via the interaction with GRK2, which may promote the heart failure (Naga Prasad et al., 2002). Thus the downregulation of Pik3ca and subsequent inhibition in the binding of GRK2 to Pik3ca by wogonin has double significance to the therapy of myocardial hypertrophy.

Wogonin downregulates Pik3ca by accelerating its degradation, since wogonin promotes the ubiquitination of Pik3a (**Figure 6C**). Nedd4l is a Pik3ca specific ubiquitin E3 ligase (Wang Z. et al., 2016) whose mRNA and protein levels were reduced by isoprenaline treatment (**Figure 7**), suggesting that isoprenaline-mediates the up-regulation of Pik3ca not only by activating its transcription (**Figure 6B**) but also by reducing its protein degradation. Wogonin enhances Nedd4l expression at the transcription level (**Figures 7C,F**), without affecting its protein stability (Figures D,E). It is worth emphasizing that we cannot exclude the possibility that other ubiquitin E3 ligases of Pik3ca are also regulated by wogonin. Additional ubiquitin ligases that potentially act on Pik3ca are still being searched.

**FIGURE 8:**
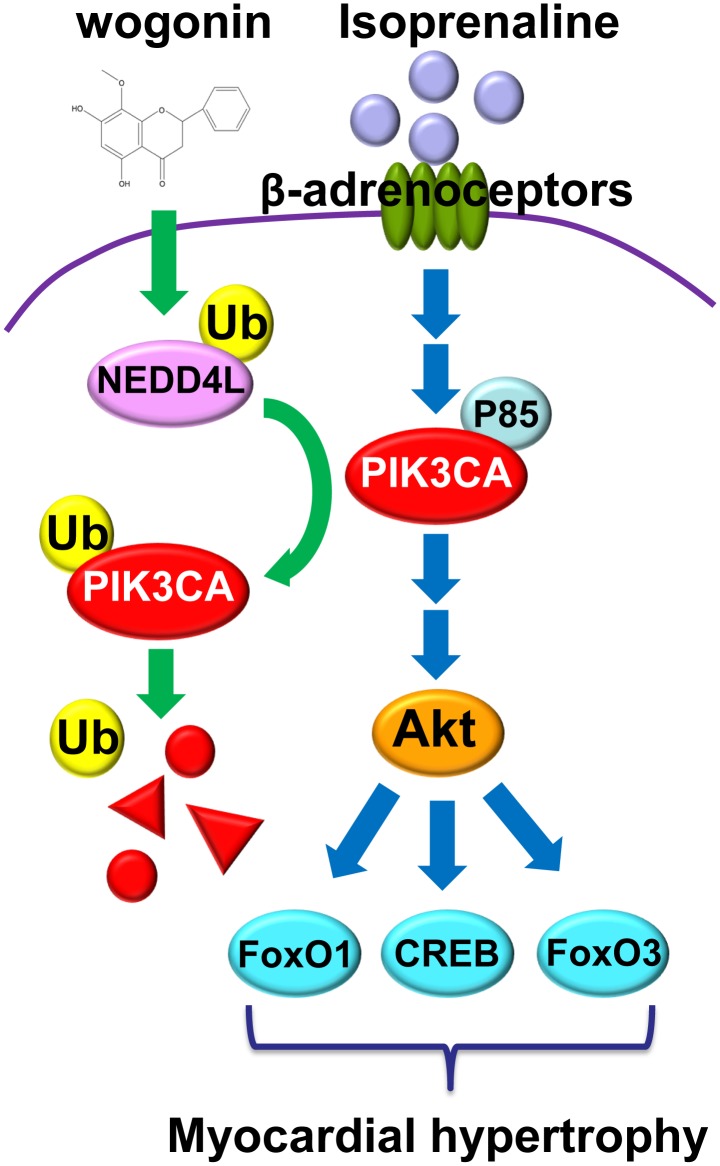
Wogonin elevates the expression of Nedd4l in cardiomyocytes, which promotes the ubiquitination and degradation of its substrate, Pik3ca.

As shown in **Figure 8**, the present investigation has revealed that wogonin targets Nedd4l and elevates its expression in cardiomyocytes, which promotes the ubiquitination and degradation of its substrate, Pik3ca. Thus, wogonin suppresses the signaling transduction in the PI3K/Akt pathway mediating the expression of hypertrophic genes, and ameliorates the myocardial hypertrophy induced by isoprenaline treatment in mice. Nowadays, β-blockers are the main drugs clinically used to treat cardiac hypertrophy and heart failure that feature over-activation of β-adrenoceptors (Khalilimeybodi et al., 2017). In the early stage of β-blocker treatment, hypofunction in the left ventricle often occurs in patients due to the suppression in the β-adrenoceptors/cAMP/PKA pathway, which would lead to intolerance. Therefore, β-blockers cannot be used in the target dose for some patients (Kukin et al., 1999). In that case, drugs to improve cardiac hypertrophy independent of the β-adrenoceptors/cAMP/PKA pathway such as wogonin, targeting the PI3K/Akt pathway, may be beneficially supplemented.

## Author Contributions

LS, MC, and WQ contributed to experiment design. WQ, DY, JZ, QH, CT, PL, XW, PY, and QL performed the experiments and analyzed the data; WQ and DY wrote the initial draft of manuscript. LS and MC reviewed the manuscript. LS and WQ obtained the funding.

## Conflict of Interest Statement

The authors declare that the research was conducted in the absence of any commercial or financial relationships that could be construed as a potential conflict of interest.

## Supplementary Material

FIGURE S1 Wogonin reduced the binding of GRK2 to Pik3ca. H9c2 Cells were treated with isoprenaline (10 μM) and/or wogonin (10 μM) as indicated for 24 h. Cell extracts were immunoprecipitated by GRK2 antibody plus protein A beads and immunoblotted with antibodies against Pik3ca and GRK2.
