# Supplementary figures and images for: Wogonin Attenuates Isoprenaline-Induced Myocardial Hypertrophy in Mice by Suppressing the PI3K/Akt Pathway
Source: Front Pharmacol. 2018 Aug 13;9:896. doi: 10.3389/fphar.2018.00896 (PMC6099096; doi:10.3389/fphar.2018.00896)

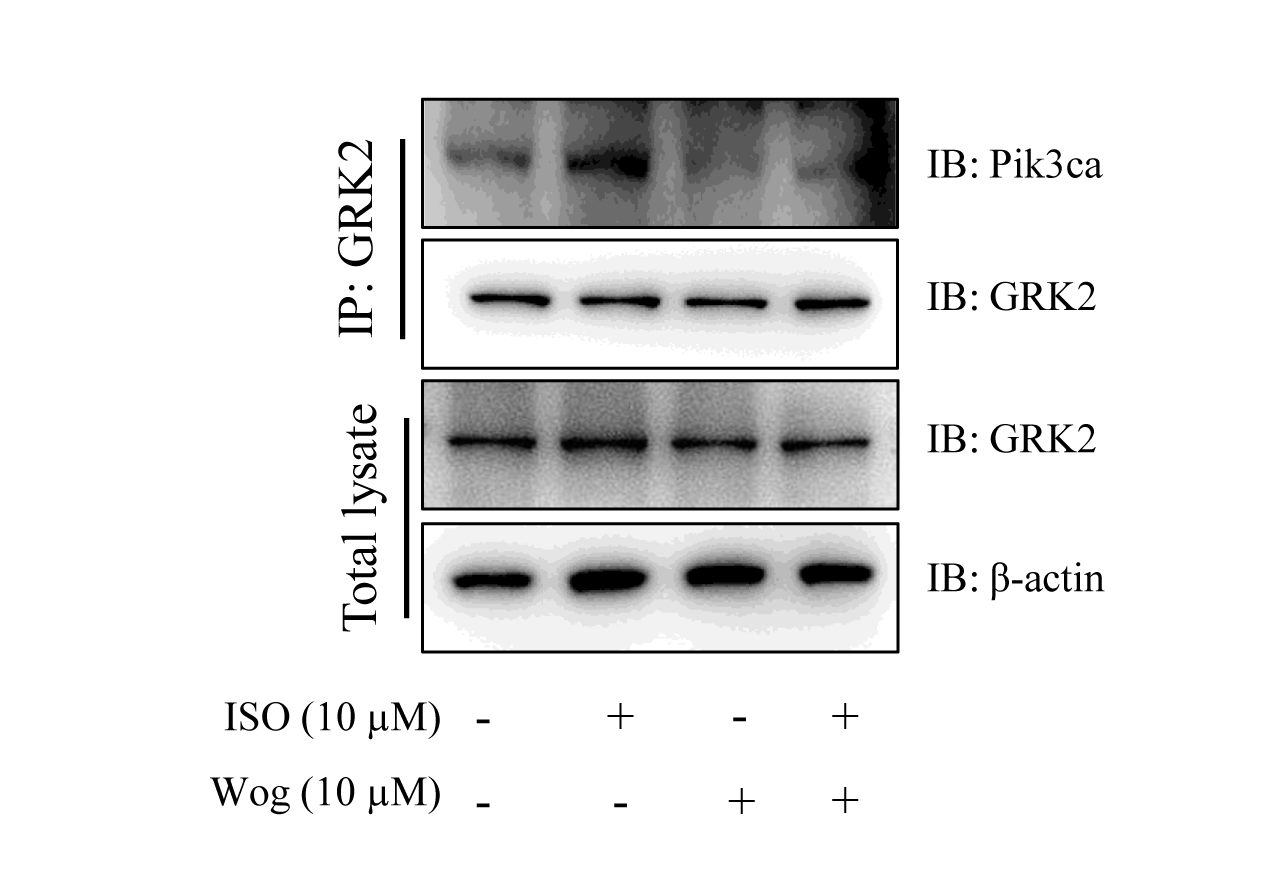

Supplement: FIGURE S1 — Wogonin reduced the binding of GRK2 to Pik3ca. H9c2 Cells were treated with isoprenaline (10 μM) and/or wogonin (10 μM) as indicated for 24 h. Cell extracts were immunoprecipitated by GRK2 antibody plus protein A beads and immunoblotted with antibodies against Pik3ca and GRK2. [file Image_1.TIF]
